# The Hospital Officer's Bookshelf

**Published:** 1912-09-14

**Authors:** 


### THE HOSPITAL OFFICER'S BOOKSHELF.

Cerebral Decompression in Ordinary Practice. By C. A. Ballance, M.V.O., F.R.C.S. (Macmillan and Co., Ltd. 1912. Pp. 71. Price 2s. 6d. net.)

This is a careful statement of the case for operative treatment of certain accidents and diseases attended by increased intra-cerebral tension, due to hæmorrhage or other causes. Among the conditions especially singled out for decompression are: Subdural hæmorrhage of the new born; fractured base; cases of *intermittent* headache, paralysis, or insanity after apparent recovery from head injury; apoplexy in the acute stage; cerebral tumour in the early stages. The author recognises that for some of these conditions lumbar puncture sometimes relieves; but seems to incline to trephining as preferable and more certain of success. His argument is illustrated by many interesting cases from his own experience, and is very ably and lucidly expounded. It is, no doubt, full value for the price charged; but as a commercial venture we believe it would be a greater success at, say, a shilling.

An Essay on Hasheesh, including Observations and Experiments. By Victor Robinson. (New York: *Medical Review of Reviews*. Price 50 cents.)

This essay is not likely to add much to our knowledge of the subject. It opens by compressing into a few pages numerous allusions to drugs, their origins and uses, and in a style horribly reminiscent of Oscar Wilde at his worst covers paragraphs, indeed pages, with names which quite obviously have been introduced for the double purpose of displaying the learning of the author and for the effect that a list of names may always be relied on to produce. Names of places and things have stirred the human spirit more than any other words in language, and this effect is here misused without stint. The observations at the end of the essay are of the ordinary kind which young men produce who take hasheesh as an experiment. They are set forth without the art of De Quincey, and with all the gravity of a man who has no sense of humour.

Materia Medica and Therapeutics. An Introduction to the Rational Treatment of Disease. By J. Mitchell Bruce, M.D., F.R.C.P., and Walter J. Dilling, M.B., Ch.B. Ninth Edition, revised. (London: Cassell and Co., Ltd. 1912. Price 6s. 6d. net.)

There must be a large number of medical men at present in active practice to whom Dr. Mitchell Bruce's little book is an old and well-tried friend. They will, doubtless, not be at all surprised to learn that the little red book still flourishes and has now attained the honour of a ninth edition. This time the author has been assisted by Dr. Walter Dilling, of Aberdeen, but the general plan of the book remains unaltered. It is, as before, chiefly therapeutical in its scope and remains one of the best introductions we possess to the rational treatment of disease, while as a guide to materia medica for the student it is unsurpassed. The many advances lately made in the physiology of the digestion and the circulation have called for considerable changes in the section on general therapeutics, and these have been made in a masterly manner. Such subjects as organotherapy and ionic medication are now touched upon in an appendix; but the greater part of this is devoted to the important matter of vaccines. This will be found a very useful introduction for the student to a subject which no class of practitioner can afford to neglect. A short description is given of a number of vaccines in respect of their preparation, dosage, and their employment. Most of the newer remedies which have proved useful in practice receive brief mention, including atoxyl and salvarsan. The Indian and Colonial Addendum is a feature which adds very greatly to the usefulness of the book for practitioners. The success of this manual in the past has been thoroughly deserved, and we are confident that it will continue to be the favourite text-book in this province of medicine for very many years to come.

SMALLPOX AND ITS DIFFUSION. By ALEXANDER COLLIE, M.B. (Bristol: John Wright and Sons. 1912. Price 2s. net.)

THIS monograph of some fifty-eight pages is devoted to the demolition of the theory of the dissemination of smallpox infection by distal aerial convection. The data upon which the author rests his case are mainly derived from his experiences as a fever hospital medical officer in the 'seventies and 'eighties of the last century. In the nature of things the argument is largely statistical, and does not lend itself to condensation. Its presentation would gain force if fewer French and Latin phrases and fewer italicised words were employed.

A TREATISE ON TREATMENT. By JOGENDER LAL CHUNDRA, L.M.S. (Calcutta. 1911. Price 9 rupees 6 annas.)

A LARGE amount of information concerning the treatment of many diseases is contained in this volume. Unfortunately the author is somewhat inclined to dot down a dozen or more methods of treatment for one condition, without any clear indication of their relative value in his opinion. Useful prescriptions abound in all directions, but so also do worthless ones. The author has in fact produced what is much more an encyclopædia than a guide. It may be added that he does not confine himself to treatment, but includes brief sketches of the symptoms and ætiology of the diseases he deals with.

AN HISTORICAL OUTLINE OF AMBULANCE. By CHARLES H. MILES, L.R.C.P. (Bristol: J. Wright and Sons. 1912. Price 3d. net.)

WE are not sure that Mr. Miles is quite correct in his use of the word "ambulance," which he apparently uses as a substantive meaning first-aid work and organisation. The substantive "ambulance," however, is already appropriated to mean a wheeled vehicle for the transference of sick or injured patients (and in military work it has a special meaning, different from this). Apart from this small point, we have nothing to criticise in this pamphlet, which traces the origin of modern ambulance work back to Homeric times. The Order of St. John of Jerusalem is, of course, dealt with; we think this section might usefully be expanded when Mr. Miles is called upon for a second edition of his little pamphlet.

ELEMENTS OF PRACTICAL MEDICINE. By ALFRED H. CARTER, M.D., M.Sc. Tenth Edition. (London: H. K. Lewis. 1912. Price 9s.)

CARTER'S *Medicine* still holds the field as an introductory text-book for medical students. Its claim to this position rests chiefly on the author's sound method of treating the subjects from the general standpoint. Unavoidably this must mean a somewhat restricted range; but, on the other hand, this is the only way in which a solid foundation can be laid on which the student can build with the bricks of experience and observation. There is no fear that the gaps and crevices will not be filled in during his fourth and fifth years in hospital. It is easy, indeed, to run through Dr. Carter's text-book and complain that such and such a treatment is omitted, or that this method is no good, and that idea is controverted by the specialists, but the fact remains that this text-book is just the very thing for the student to read, mark, learn, and inwardly digest until he begins to feel his feet. He will be quite safe if he neglects larger text-books for the first year after his second examination is behind him, for Dr. Carter provides him with a very satisfying diet. This is no "cram" book, no indigestible condensation, but a thoroughly excellent and not too elementary text-book from which he may learn a great deal more than from many others he may read.

A PRACTICAL MEDICAL DICTIONARY. By THOMAS LATHROP STEDMAN, A.M., M.D. (London: Henry Frowde and Hodder and Stoughton. 1911. Pp. 1,000. Illustrated. With Thumb Index. Price 21s.)

WE may well believe that the compilation of this dictionary was the labour of many years; it contains about 50,000 references, and, as far as can be gathered from a necessarily casual examination, there are extremely few errors, typographical or otherwise. Etymology is a strong point in this dictionary and increases its usefulness enormously. As regards the vocabulary, the author has endeavoured to throw the weight of whatever authority may be accorded to him on to the side of strictly correct etymology. Naturally this in many cases conflicts with common usage. Some of our most familiar and cherished terms are, no doubt, as Dr. Stedman says, "barbaric and cacophonic mixtures, painful to the ear and vexatious to the spirit of anyone with a sense of linguistic fitness." The latter quality not being one which, we fear, troubles the average member of the medical profession, *appendicitis* will probably survive the claims of *scolecoiditis*. Special attention has been paid to synonyms and cross references are numerous. In this dictionary terms in allied sciences which medicine touches at many points are included. An effort has been made to allow of reference to the chemical, botanical, dental, and veterinary words which a physician is likely to meet in his reading, while a large number of definitions of the terms relating to insurance, life tables, policies, etc., have been introduced. Under the titles of artery, nerve, muscle, duct, etc., are given lists of these in alphabetical order. The Basle anatomical nomenclature is given the preference in all cases, but the older terms are also referred to. A large number of eponymic terms will be found under the proper name, *e.g.*, *Hirschsprung's disease* under H and not under *disease*; this is more useful for reference, and gives, moreover, the opportunity for the insertion of brief biographical details regarding nationality, dates of birth, death, etc. In the spelling of certain words the author has preferred to eliminate the diphthongs *æ*, *œ*, on the ground that the direct descendants of Latin, Italian, and Spanish have dropped the *a* and the *o* and that in English we write *equal* and *economy*. But he also does the same for words containing Greek diphthongs, so that it is difficult to draw the line. A brave and unfortunately almost necessary concession has been made in the transliteration of Greek words into Roman characters. Dr. Stedman strongly protests against the elimination of Greek in our schools, and is of opinion that this has done much to barbarise the language of medicine. We trust, however, that the percentage of even well-educated physicians of the present day who read Greek characters is not so small as the author imagines. This dictionary is a very worthy example of the practical lexicon, it is handy in size, has a useful thumb index, and is well bound. If any criticism is warranted it would concern the illustrations, which are wasters of valuable space and in themselves of scarcely any merit.

